# Successful Use of Neurally Adjusted Ventilatory Assist in Managing Refractory Bilateral Pneumatoceles in a Premature Neonate

**DOI:** 10.1055/a-2945-0611

**Published:** 2026-09-02

**Authors:** Efren Diaz, Shamaila Gil, Lisa M. Scheid

**Affiliations:** 1 Division of Neonatal-Perinatal MedicineDepartment of Pediatrics 12334 University of Texas Southwestern Medical CenterDallasTexasUnited States

**Keywords:** pneumatoceles, neonates, NAVA, ventilation, pneumonia, prematurity, air leak

## Abstract

**Background** Pneumatoceles are air-filled cystic lesions within the lung parenchyma, often arising from severe pneumonia or mechanical ventilation-induced lung injury, which pose significant risk, particularly in neonates requiring high positive-pressure ventilation. Traditional management includes conservative monitoring, percutaneous drainage, and/or surgical intervention. High-frequency ventilation is frequently employed to minimize barotrauma.

**Case Summary** We present a neonate born at 29 ^0/7^ weeks of gestation, weighing 780 g. Post-delivery, the patient developed pneumonia, leading to multiple refractory bilateral pneumatoceles and transfer to our hospital. Initial management included high-frequency jet ventilation and inhaled nitric oxide, with limited improvement over 3 weeks, after which we initiated neurally adjusted ventilatory assist (NAVA), leading to significant clinical improvement. Radiographic imaging showed rapid improvement of the pneumatoceles on NAVA, with complete resolution observed at outpatient follow-up.

**Discussion** This case demonstrates successful management of refractory bilateral pneumatoceles using NAVA. Its patient-driven approach provides synchronized, gentle ventilation, crucial for treating pulmonary air leak syndromes and facilitating natural weaning. NAVA’s dynamic adjustments and ability to closely match ventilatory support to the patient’s neural respiratory drive using less peak inspiratory pressure offers a promising strategy for managing complex pulmonary conditions, such as pneumatoceles, in neonates.

## Background

Pneumatoceles are thin-walled, air-filled cystic lesions that develop within lung parenchyma. They most commonly develop as a sequela of severe pulmonary infections or as a consequence of mechanical ventilation–induced lung injury. 1 2 3 The physiologic mechanism involves a check-valve effect, whereby air enters the cystic space but is unable to escape, leading to progressive overdistension. 4 Bacterial etiologies predominate, with *Staphylococcus aureus* being the most frequently implicated pathogen. However, organisms such as *Streptococcus pneumoniae* , *Escherichia coli* , *Klebsiella pneumoniae* , *Enterobacter cloacae* , and *Pseudomonas aeruginosa* have also been associated with this pathology. 2 4 5 6 Although less common, gram-negative pneumonias—particularly those caused by *Acinetobacter calcoaceticus* —have been observed, notably in patients who require high levels of positive-pressure ventilation. 6 The risk of pneumatoceles is further elevated with mechanical ventilation, especially when employing high positive end-expiratory pressure (PEEP), as this can induce alveolar overdistension. 1 3 Diagnosis relies primarily on imaging modalities; chest radiographs typically reveal characteristic thin-walled cystic lesions, while computed tomography (CT) provides a more detailed assessment, delineating the size, location, number of lesions, and any associated complications such as pneumothorax or abscess formation. 1 4 7

Management strategies for pneumatoceles are tailored to the severity of symptoms and the presence of complications. Many lesions can be managed with conservative monitoring and supportive care using lower mean airway pressure (MAP) with conventional ventilation, and will resolve spontaneously. High-frequency ventilation is commonly employed, utilizing rapid, small volume breaths to maintain lung inflation and improve gas exchange while minimizing the risk of barotrauma. 1 8 9 10 However, in instances where significant respiratory compromise occurs or complications such as tension pneumatoceles or abscess formation develop, more invasive interventions may become necessary. Therapeutic options include percutaneous catheter drainage, selective bronchial intubation, and, in severe cases, surgical resection. 1 5 8 11 In complex cases, video-assisted thoracoscopic surgery has also been employed to facilitate direct visualization and effective drainage. 12

The prognosis for patients with pneumatoceles varies depending on the underlying cause, the size and number of the lesions, and the presence of any complications. Many neonates and infants with pneumatoceles recover completely with resolution of cysts within weeks to months. 1 5 8 11 However, patients with severe infections or those requiring prolonged mechanical ventilation may have a more complicated course, with an increased risk of developing bronchopulmonary dysplasia or other long-term pulmonary sequelae. 13

### Neurally Adjusted Ventilatory Assist

Neurally adjusted ventilatory assist (NAVA) is an advanced form of mechanical ventilation that utilizes a patient’s own diaphragmatic electrical activity (Edi) to adjust the level of ventilatory support. This technique ensures a high degree of synchronization between the patient’s neural respiratory drive and the ventilator’s assistance.

The initiation of spontaneous respiration is governed by the central respiratory centers, which transmit efferent signals via the phrenic nerves to the diaphragm, prompting its contraction. 14 This neuromuscular activation results in diaphragmatic descent, generating negative intrathoracic pressure and facilitating alveolar ventilation. The amplitude and pattern of diaphragmatic electrical activity are directly proportional to the central respiratory drive and effort. In NAVA, a specialized nasogastric or orogastric catheter embedded with multiple electrodes is positioned at the esophagogastric junction to detect the Edi signal with high temporal resolution. The ventilator then delivers inspiratory pressure in direct proportion to the detected Edi amplitude, ensuring that the support aligns precisely with the patient’s inspiratory effort. 14 15 As a result, several studies have shown using NAVA allows for decreased peak inspiratory pressure compared with other forms of mechanical ventilation, including synchronized pressure-controlled and volume-controlled ventilation. 16 17 This proportional assistance continues throughout the inspiratory phase and terminates in synchrony with the cessation of diaphragmatic activity, thereby closely mimicking natural breathing patterns. 18 This form of electrical synchronization is much more sensitive than traditional flow sensors, thereby minimizing patient–ventilator asynchrony, which can often be associated with ventilator-induced lung injury, such as pneumothorax, and prolonged mechanical ventilation. 14 15 18 19

NAVA has shown several clinical benefits, including improved patient–ventilator interaction, preservation of diaphragmatic function, and individualized ventilatory support. By aligning assistance with the patient’s intrinsic drive, NAVA not only reduces asynchrony, but has also been shown to decrease the need for sedation. 14 20 21 In addition, continuous monitoring based on Edi helps prevent diaphragmatic atrophy associated with controlled ventilation modes. 22 Lastly, NAVA allows for dynamic adjustment of ventilatory support in response to changing respiratory demands, which is particularly beneficial for populations with variable breathing patterns, such as neonates. 19

### Case Presentation

We present a case of an extremely premature and very low birth weight female, born via C-section to a 31-year-old G4P2 mother with negative maternal serologies, at 29 ^0/7^ weeks of gestation and weighing 780 g at birth. The pregnancy was complicated by intrauterine growth restriction and pre-eclampsia, prompting a premature delivery after maternal administration of antenatal steroids. APGAR scores were 8 and 9 at 1 and 5 min of life, respectively; however, the infant required immediate respiratory support at birth via bubble continuous positive airway pressure (CPAP) with prompt escalation to intubation on high-frequency jet ventilation (HFJV) and surfactant administration for persistent hypoxemia. At day 11 of life, the patient developed Methicillin-sensitive *Staphylococcus aureus* pneumonia complicated by multiple bilateral pneumatoceles. Further complications over the first month of life included acute renal failure in the setting of a patent ductus arteriosus, necessitating treatment with a course of acetaminophen. After a course of dexamethasone, the patient was eventually extubated back to CPAP, but lack of improvement of the bilateral pneumatoceles prompted a transfer to our facility for a higher level of care.

**Fig. 1:**
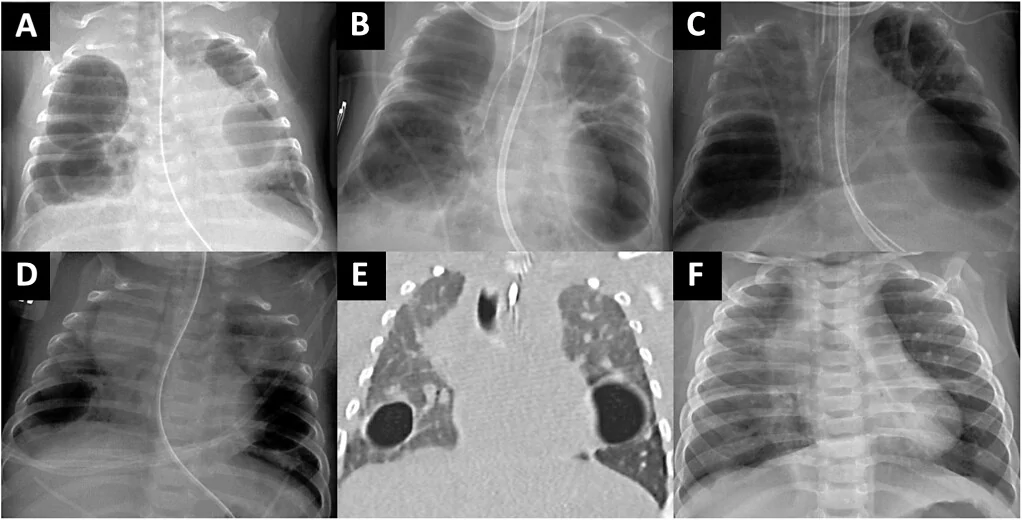
Stages of pneumatoceles during management. ( **A** ) Day of hospitalization at our neonatal intensive care unit (HD) 1/day of life (DOL) 36: Bilateral pneumatoceles on high-frequency jet ventilation (HFJV). ( **B** ) HD 21/DOL 56: Bilateral pneumatoceles on HFJV. ( **C** ) HD 22/DOL 57: Bilateral pneumatoceles on neurally adjusted ventilatory assist (NAVA) for 1 day. ( **D** ) HD 67/DOL 102: Bilateral pneumatoceles on low flow nasal canula (NC; ≤1 L/min). ( **E** ) HD 74/DOL 109: Bilateral pneumatoceles on NC on chest computed tomography (CT). ( **F** ) Outpatient follow-up on room air/no respiratory support (RA) at 8 months of age.

Upon arrival to our neonatal intensive care unit (NICU), the patient presented with worsening respiratory acidosis and severe hypoxemia prompting re-intubation and placement on HFJV (Bunnell Life Pulse, Salt Lake City, Utah, United States). Of note, the patient was previously extubated only 1 day prior to transfer. Radiographic images of diffuse pneumatoceles at the time of arrival to our NICU can be seen in Fig. 1A . Initial ventilator settings included a peak inspiratory pressure (PIP) of 26, PEEP of 8, and frequency of 360 breaths per minute, though required adjustment to a PIP of 38, PEEP of 15, and frequency of 240, with an MAP of 18 to achieve stabilization. Lower respiratory cultures grew *Pseudomonas aeruginosa.* Respiratory support was adjusted daily based on radiographic evidence of lung expansion and blood gases. Multiple attempts to wean to lower PEEP and MAP while on HFJV resulted in profound hypoxemia with saturations in 60s on 100% FiO _2_ and hypercapnia with CO _2_ in 70s to 90s, necessitating stabilization at higher pressures. Despite intensive care over a 3-week span, there was negligible improvement in gas exchange and pneumatocele size as assessed by radiographic imaging. On day 21 at our NICU, the decision was made to attempt NAVA (MAQUET Servo-i; Getinge, Goteborg, Sweden) given the lack of respiratory progress ( Fig. 1B ) and the fact that the presence of multifocal disease complicated surgical intervention, increasing both technical complexity and the risk of morbidity and mortality. Initial settings were a NAVA level of 1.8, PEEP 12, PS 10, Edi trigger of 0.5, apnea time of 30 seconds, and a backup rate of 30. Within 24 hours, the PEEP was weaned to 7 with MAP of 11, and there was a significant decrease in pneumatocele size noted on radiographic imaging ( Fig. 1C ). Within 17 days, the patient was extubated to bubble CPAP with PEEP of 8 and was on 4 L of high-flow nasal canula 2 days later. Over the course of the next 2 weeks, the patient was weaned to 1 L of low-flow oxygen via nasal canula and eventually discharged home on 0.25-L nasal canula oxygen at day of life 115 after 80 days of hospitalization at our NICU. Roughly 6 weeks after discharge, the patient was taken off home oxygen support.

**Fig. 2:**
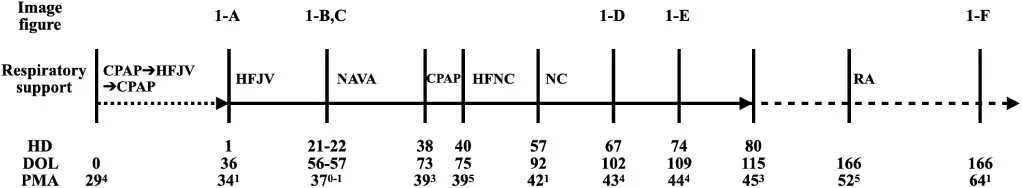
Timelines of events. The short dashed line indicates hospitalization at an outside neonatal intensive care unit (NICU). The solid line denotes hospitalization at our NICU. The long dashed line denotes outpatient follow-up. Images correspond to the radiographic images in Fig. 1 . Respiratory support changes are denoted by the preceding vertical time marker. Postmenstrual age (PMA) is denoted in weeks. Superscript numbers denote the number of days in an incomplete week. CPAP, continuous positive airway pressure; DOL, day of life; HD, day of hospitalization at our NICU; HFJV, high-frequency jet ventilation; HFNC, high-flow nasal canula; NAVA, neurally adjusted ventilatory assist; NC, low-flow nasal canula (≤1 L/min); RA, room air/no respiratory support.

Regarding the pneumatoceles size, we observed expansion of the cystic structures with shifting atelectasis consistently while on HFJV. Once on NAVA, we began to see significant improvement in bilateral upper lobe pneumatoceles within 24 hours ( Fig. 1C ) and complete resolution of the right upper lobe pneumatocele within a week ( Fig. 1D ). CT scan 1 month later demonstrated further improvement of all pneumatoceles ( Fig. 1E ) with resolution of major cystic lesions seen on outpatient follow-up at 8 months of age ( Fig. 1F ). A complete timeline of hospitalization events including respiratory support and imaging can be seen in Fig. 2 . Of note, head ultrasound and brain magnetic resonance imaging completed during hospitalization showed no evidence of intraventricular hemorrhage though they did show mild atrophy of bilateral cerebellar hemispheres and brainstem for which the patient was referred to neurology and early childhood intervention.

## Discussion

This case utilized NAVA to successfully treat refractory bilateral pneumatoceles and offers insight into NAVA as a rescue therapy. While traditional pneumatocele management often focuses on conventional ventilation with low MAP, not all patients tolerate such strategies, as was demonstrated in this case. Because NAVA delivers support proportional to the patient’s effort, it proves a gentler form of ventilation utilizing lower peak inspiratory pressures compared with traditional pressure-control or volume-control modes of ventilation. This was very evident with our patient as ventilator pressures were able to be significantly weaned within 24 hours of NAVA initiation. This approach is advantageous for managing fragile lung tissues affected by pneumatoceles or other air leak syndromes where there is heterogenous compliance. The dynamic adjustments offered by NAVA are critical in managing fluctuating respiratory needs without causing excessive pressure or volume delivery, which could otherwise worsen pneumatoceles. In our patient, the dynamic and responsive adjustments provided by NAVA promoted rapid radiographic improvement of the pneumatoceles in less than 1 day. By preventing the overdistension of alveoli and providing synchronized support, NAVA helps reduce the incidence and severity of air leak syndromes.

In addition to its lung-protective advantages, the patient-driven nature of NAVA allows for smoother transition toward weaning and extubation. As the patient’s respiratory effort decreases, the support provided by NAVA diminishes accordingly. This is particularly important for preterm infants with pneumatoceles, as prolonged mechanical ventilation and barotrauma can lead to further complications, such as bronchopulmonary dysplasia.

NAVA not only mitigated the deleterious effects of positive-pressure ventilation but also redefined our approach to weaning by ensuring that ventilatory support weaned in conjunction with our patient’s improving respiratory drive. This synchronization is paramount in preventing further lung injury, optimizing gas exchange, and ultimately improving long-term pulmonary outcomes.

In conclusion, our experience with NAVA in this challenging case underscores its potential as a rescue strategy in the management of complex neonatal pulmonary conditions, such as pneumatoceles. As we continue to seek optimal modalities that balance respiratory support with lung protection, NAVA remains a promising tool that could redefine ventilatory management protocols for our most challenging clinical situations. Future studies are warranted to validate these findings and to explore the broader applicability of NAVA in neonatal respiratory failure.

## Data Availability

No datasets were generated or analyzed during the current study. All relevant information is included within the article.

## Declaration of GenAI Use

The authors confirm that no Generative AI was used in the preparation of this manuscript.
